# Detection of multidrug-resistant bacteria in the nasal cavities and evaluation of sinus disorders in patients undergoing Le Fort I osteotomy

**DOI:** 10.1186/s12903-024-04295-6

**Published:** 2024-05-04

**Authors:** Bárbara M. Mendes, Évelin S. Bortoli, Catherine B. Zaleski, Maila P. D. Martinelli, Vanessa F. Pascoal, Sílvia D. Oliveira

**Affiliations:** 1 Laboratório de Imunologia e Microbiologia, Escola de Ciências da Saúde e da Vida, Pontifícia Universidade Católica do Rio Grande do Sul, PUCRS, Av. Ipiranga, 6681, Porto Alegre, RS 90619-900 Brazil; 2 Programa de Pós-graduação em Odontologia, Escola de Ciências da Saúde e da Vida, Pontifícia Universidade Católica do Rio Grande do Sul, PUCRS, Porto Alegre, RS Brazil

**Keywords:** Orthognathic surgery, Le Fort I, Sinus disorders, Maxillary sinus, *Klebsiella pneumoniae*, Antimicrobial resistance

## Abstract

**Introduction:**

Orthognathic surgery can lead to sinus alterations, including sinusitis, attributed to the exposure of maxillary sinuses during Le Fort I osteotomy. Furthermore, being a hospital-based procedure, there is potential risk of complications arising from bacteria prevalent in such environments. This study evaluated maxillary sinusitis occurrence and the presence of multidrug-resistant bacteria in the nasal cavity before and after orthognathic surgery.

**Methods:**

Ten patients with dentofacial deformities underwent Le Fort I osteotomy. Clinical evaluations using SNOT-22 questionnaire were performed, and nasal cavity samples were collected pre-surgery and 3–6 months post-surgery to quantify total mesophilic bacteria and detect *Staphylococcus aureus*, *Acinetobacter baumannii*, and *Klebsiella pneumoniae*. Cone Beam Computed Tomography (CBCT) was performed pre- and post-operatively, and the results were evaluated using the Lund-Mackay system. This study was registered and approved by the Research Ethics Committee of PUCRS (No. 4.683.066).

**Results:**

The evaluation of SNOT-22 revealed that five patients showed an improvement in symptoms, while two remained in the same range of interpretation. One patient developed post-operative maxillary sinusitis, which was not detected at the time of evaluation by SNOT-22 or CBCT. CBCT showed a worsening sinus condition in three patients, two of whom had a significant increase in total bacteria count in their nasal cavities. The Brodsky scale was used to assess hypertrophy in palatine tonsils, where 60% of the subjects had grade 1 tonsils, 20% had grade 2 and 20% had grade 3. None of the patients had grade 4 tonsils, which would indicate more than 75% obstruction. Two patients harboured *S. aureus* and *K. pneumoniae* in their nasal cavities. Notably, *K. pneumoniae*, which was multidrug-resistant, was present in the nasal cavity of patients even before surgery, but this did not result in maxillary sinusitis, likely due to the patients’ young and healthy condition.

**Conclusion:**

There was an improvement in signs and symptoms of maxillary sinusitis and quality of life in most patients after orthognathic surgery. However, some patients may still harbour multidrug-resistant bacteria, even if they are asymptomatic. Therefore, a thorough pre-operative assessment is essential to avoid difficult-to-treat post-operative complications.

## Introduction

Orthognathic surgery (OS) encompasses a wide range of procedures involving osteotomies of the maxilla and/or mandible and is the standard approach to correcting congenital, developmental, and acquired dentofacial discrepancies, thereby improving occlusion, facial aesthetics and the management of airway obstruction [1, 2]. Typically, the Le Fort I osteotomy is one of the primary techniques employed in maxillary OS [3]. In this procedure, a horizontal incision is made above the apices of the maxillary teeth, starting from the nasal rim and extending posteriorly to the zygomatic buttress. This incision passes through the walls of the maxillary sinuses and the nasal septum on both sides [1]. The Le Fort I osteotomy is often chosen because of its short duration, simple anatomical approach, consistent long-term outcomes, and adaptable method of repositioning the maxilla in three-dimensional space [4].

The most common complication of OS is intraoperative and post-operative bleeding, which can be life-threatening [4]. Moreover, surgical alterations to the anatomy and physiology of the maxillary sinuses may lead to sinus inflammation and post-operative infection [5]. Although the maxillary sinus microbiota has been described to be highly variable among individuals [6, 7], an overall proportion (24.5 ± 10.6%) of stable taxa was observed in subjects with longstanding maxillary antrostomies at different time points [8]. Certain microbial taxa, such as *Staphylococcus*, *Corynebacterium*, *Propionibacterium*, and *Pseudomonas*, were consistently present but fluctuated in relative abundance. Conversely, *Janthinobacterium*, *Enterobacter*, *Lactobacillus*, and *Acinetobacter* were prevalent and moderately abundant in healthy sinuses, but not in inflamed sinuses. *Moraxella* and *Haemophilus*, on the other hand, were less frequently present and proportionally less abundant in sinuses experiencing chronic or intermittent inflammation compared to healthy sinuses [8]. *Staphylococcus* spp. have also been found to be prevalent in the sinus cavities of patients with chronic rhinosinusitis, with *Staphylococcus aureus* being the predominant species [9–13]. Some studies address that aerobic bacteria are relevant in both chronic and acute infections, while anaerobic bacteria are predominant in chronic infections of odontogenic origin [14–16].

Considering that Le Fort I osteotomy is performed in a hospital environment, the most frequent bacteria associated with healthcare-related infections become an important concern as potential post-operative complications. Among these, *Acinetobacter baumannii*, *Klebsiella pneumoniae*, and *S. aureus* stand out as opportunistic and often multidrug-resistant bacteria that may lead to therapeutic failure [17]. In this context, the aim of this study was to analyze the occurrence of maxillary sinusitis in patients undergoing OS with Le Fort I osteotomy and to determine the presence of the *A. baumannii*, *K. pneumoniae* and *S. aureus*, as well as their susceptibility to antibiotics.

## Materials and methods

### Participants

The study was designed as a clinical, observational, analytical, longitudinal, and prospective investigation involving 10 patients. It adhered to the ethical standards of the Declaration of Helsinki and was approved by the Research Ethics Committee of the PUCRS (No. 4.683.066).

Patients of both sexes over 18 years of age with dentofacial deformities, skeletal pattern class II or III, skeletal discrepancies, or significant asymmetries who had undergone Le Fort I osteotomy for OS were recruited. Patients were included if they did not have symptoms of sinusitis at the time of the initial evaluation at the Bucomaxillofacial Surgery and Traumatology Service of the School of Health and Life Sciences of PUCRS and at the Hospital São Lucas of PUCRS. Patients who agreed to participate in the study signed the free and informed consent form.

Exclusion criteria included buco-sinus contact due to tooth extraction, presence of implants or foreign bodies in the maxillary sinus prior to surgery; teeth close to the maxillary sinus with periodontal, periapical injuries, cysts, or overturned endodontic; history of surgery to correct facial trauma involving the third middle of the face (including the maxillary sinus); history of surgery to lift the maxillary sinus; presence of cleft lip and palate; smoking; and use of antibiotics within 4 weeks prior to OS.

### Clinical evaluation

Patients were assessed for the presence or absence of symptoms indicative of maxillary sinusitis before and after of OS and post-operatively, using tests such as levels of nasal congestion, nasal discharge, facial pain, hyposmia, and fatigue. Patients also completed a validated questionnaire for clinical assessment of symptoms of maxillary sinusitis called SNOT-22 (Sino-Nasal Outcome Test). The pre-operative questionnaire was administered during the pre-operative consultation, and the post-operative questionnaire was given at the follow-up consultation 5 and 11 months after surgery.

The SNOT-22 consists of 22 questions about nasal, paranasal, psychological and sleep-related symptoms, providing a measure of quality of life for people with sinonasal diseases. The questions are rated on a scale of 0 to 5, where 0 is no problem and 5 is the worst possible problem. The final score can range from 0 to 110, with a score of 0 to 10 indicating no or minimal problems; 11 to 40 indicating mild to moderate problems; 41 to 70 indicating moderate to severe problems, and a score above 70 indicating severe or critical conditions that may require the attention of a specialist for possible surgical intervention [5].

Palatine tonsils were scored for hypertrophy using the Brodsky scale, which grades the degree of airway obstruction. Grade I indicates obstruction of less than 25% of the airway, grade II from 25 to 50%, grade III from 50 to 75%, and grade IV more than 75%. Patients with grade III and IV palatine tonsils were considered to have tonsillar hypertrophy [18, 19].

Patient data, including age, sex, comorbidities, allergies, current medications, history of chronic sinusitis, surgical and dental interventions were collected. For patients with class II or III skeletal patterns, dentofacial deformities were analysed by clinical examination (facial analysis) and orthodontic documentation such as photographs, study models, radiographs and cephalometry.

### Tomographic evaluation

Prior to surgery, patients underwent cone beam computed tomography (CBCT) up to 30 days pre-operatively. Another CBCT was performed in the late post-operative period, from 3 to 12 months, to assess changes in the maxillary sinuses after Le Fort I osteotomy, including masking, mucosal thickening, and the presence of foreign bodies, by comparing pre- and post-operative findings. The Lund-Mackay system was used to score the findings based on the degree of opacification of the maxillary sinuses in CBCT. It should be noted that this study focused on the maxillary sinuses only.

### Bacterial evaluation of the nasal cavity

#### Sample collection

Each of the 10 patients underwent two collections, one before surgery and the other 3 to 6 months after surgery. The collections were performed using two sterile swabs in the right nasal cavity. One swab was inoculated in 1 mL of 0.85% saline solution and the other in 1 mL of Brain Heart Infusion (BHI) broth, and microbiological analyses were performed on both samples.

#### Quantification of culturable mesophilic aerobic bacteria

The saline swab was used to quantify the culturable mesophilic aerobic bacteria. To do this, the 0.85% saline solution tube containing the swab (designated “0”) was vigorously mixed using a homogenizer vortex and diluted to 10^− 2^ in 0.85% saline. A volume of 100 µL of “0” and each dilution was then spread on the surface of standard agar plates for counting, in triplicate. The plates were then incubated at 37 °C for 48 h, after which the number of colony-forming units (CFU)/mL was determined.

#### Detection of *Acinetobacter* spp., *K. pneumoniae* and *S. aureus*

The swab in BHI broth was used to inoculate MacConkey agar and salt mannitol agar surfaces, which were incubated at 37 °C for up to one week. Colonies that were compatible with *Acinetobacter* spp. and *K*. *pneumoniae* on MacConkey agar, and with *Staphylococcus* spp. on salt mannitol agar, were grown in BHI broth at 37 °C overnight and stored at -20 °C with the addition of 30% glycerol (v/v) for further identification. Isolates were identified by matrix-assisted laser desorption ionization time of flight (MALDI-TOF) mass spectrometry (Microflex LT, Bruker Daltonik®, Bremen, Germany), according to Hijazin et al. [20], at the Instituto Adolfo Lutz, São Paulo, Brazil. Bacteria were grown on trypticase soy agar or nutrient agar (Oxoid, Basingstoke, UK) at 30 °C for 20–24 h. The culture (1 mL) was then centrifuged at 5,000 rpm for 5 min, the pellet was treated with 70% ethanol, and then the mixture was centrifuged at 13,000 rpm for 2 min. The proteins were then precipitated, placed on a steel plate, and covered with the matrix (10 mg/mL of α-cyano-4-hydroxy-cinnamic acid in 50% acetonitrile/2.5% trifluoroacetic acid). Each bacterial isolate was spotted into three wells, and three reads were performed for each plate. FlexControl software (Bruker Daltonik) was used to acquire protein spectra using the MTB_autoX method with a mass range from 2 to 20 kDa. BioTyper 3.0 software (Bruker Daltonik®) was used to analyse the mass spectra by comparison with the profiles stored in the system library. The criteria for interpreting the standards used were those of the manufacturer, as follows: scores ≥ 2.0 were accepted for species assignment, and scores ≥ 1.7 and < 2.0 were used for genus identification only.

#### Assessment of bacterial susceptibility to antibiotics

Isolates identified as *K*. *pneumoniae* and *S. aureus* were evaluated for antibiotic susceptibility using the disk diffusion method according to the guidelines of the European Committee on Antimicrobial Susceptibility Testing (EUCAST) [21]. *Escherichia coli* ATCC 25922 and *S. aureus* ATCC 29213 were used as reference strains for quality control during the analyses.

### Statistical analysis

Comparisons between nasal CFU counts before and after Le Fort I osteotomy were performed using paired *t*-test. Values of *P* < 0.05 were considered significant. Statistical analysis was performed using GraphPad Software Prism version 9.3.1 (GraphPad Software).

## Results

The sample consisted of 10 patients, 5 females and 5 males, with a mean age of 26.5 ± 9.5 years. The majority of individuals (80%) had a facial pattern characterised by class III skeletal deformities, while the remaining patients had class II skeletal deformities. None of the patients reported smoking, but some reported consumption of alcoholic beverages. Of the 10 patients, 3 reported having asthma and/or rhinitis, while 1 reported having diabetes and taking metformin hydrochloride. In addition, 2 patients reported taking sertraline and escitalopram as antidepressants. Two patients had a history of appendectomy, 1 of cholecystectomy and 1 of inguinal hernioplasty (Table 1).

**Table 1:** General characteristics of the patients

Patient number	Sex	Age	Skeletal angle	Smoking	Alcoholic beverages	Comorbidity
1^a^	male	27	3	no	Yes	No
2^b^	male	23	3	no	No	Asthma
3	male	17	3	no	No	No
4	female	20	3	no	No	No
5^b, c^	male	29	3	no	No	asthma – rhinitis
6^d, e^	female	25	2	no	No	No
7	female	25	3	no	No	No
8	female	28	3	no	no	asthma - rhinitis
9	female	36	3	no	No	No
10^f^	male	35	2	no	No	diabetes

^a^History of inguinal hernioplasty

^b^History of appendectomy

^c^Use of sertraline

^d^Use of escitalopram

^e^History of cholecystectomy

^f^Use of metformin hydrochloride

The evaluation of the pre-operative SNOT-22 questionnaire showed that 1 subject (10%) had no or minimal problems, 5 (50%) mild to moderate, 2 (20%) moderate to severe, and 2 (20%) severe or critical conditions that required the attention of a specialist, for a possible surgical intervention. On the other hand, the answers to the same questionnaire in the post-operative period showed that only 1 subject (10%) had moderate to severe symptoms of sinusitis and problems related to this condition, and the other patients (90%) had mild to moderate symptoms.

A comparison of the results of the SNOT-22 questionnaire before and after surgery showed an improvement in sinusitis symptoms and related problems. Specifically, 5 individuals (50%) reported an improvement in symptoms, 2 (20%) reported no change, and 3 (30%) reported a higher score, indicating a worsening of the clinical condition. However, of these 3, 2 remained within their pre-operative symptom range, and only one had an increase in symptom severity. Of the 5 (50%) patients with improved symptoms and quality of life, 3 remained in the same symptom classification range, while 2 experienced a significant improvement in their symptom classification. These findings are summarized in Table 2.

**Table 2:** Pre- and post-operative SNOT-22 scores and grade of palatine tonsil hypertrophy

Patient number	Pre-operative SNOT-22 score	Post-operative SNOT-22 score	Palatine tonsil hypertrophy
P1	15	13	1
P2^a^	43	63	3
P3	37	15	2
P4	78	17	1
P5	8	15	1
P6	65	42	1
P7	17	17	2
P8	39	17	1
P9	74	12	1
P10	20	31	3

^a^Patient with hypertrophy of palatine tonsils and high scores in pre- and post-operative SNOT-22

The subjects reported several problems and symptoms prior to undergoing surgery, as indicated by their responses to the SNOT-22 questionnaire. These included fatigue or tiredness during the day, lack of restful sleep, nasal obstruction, and post-nasal discharge. After surgery, the most common problems were the need to blow the nose, post-nasal discharge, daytime fatigue or tiredness, and frustration, restlessness or irritability.

Only one participant had symptoms of sinusitis in the post-operative period among all the participants. The patient experienced two episodes of sinonasal disease, the first of which occurred 30 days after the surgery and resolved spontaneously, without medication. The second episode occurred approximately 60 days after surgery, and was treated with a combination of amoxicillin (500 mg) and potassium clavulanate (125 mg), administered every 8 h, for 7 days, resulting in complete resolution of the problem without recurrence. Based on the pre- and post-operative SNOT-22 results and the patient’s clinical condition, it can be concluded that the episodes of sinonasal disease were isolated and not indicative of a chronic condition, as indicated by the mild to moderate symptom and problem (scores of 15 and 13, pre- and post-operative, respectively). It is important to emphasize that the patient did not show symptoms of sinusitis on the SNOT-22 questionnaire, and the diagnosis was made by clinical evaluation based on the presence of nasal congestion, yellow/green nasal discharge, headache, and facial pain.

The palatine tonsils of the participants were assessed for hypertrophy by clinical examination using the Brodsky scale to determine if they were hypertrophied. A total of 60% of the subjects had grade 1 tonsils, 20% had grade 2 tonsils and 20% had grade 3 tonsils. None of the patients had grade 4 tonsils, which would indicate more than 75% obstruction. Grades 3 and 4 are considered to be tonsil hypertrophy, therefore, in this sample, only 2 (20%) of the subjects presented with this condition (Table 2).

Pre- and post-operative tomograms were analysed using the Lund-Mackay scale to evaluate the maxillary sinuses, and changes indicating a worsening of post-operative results were observed in patients 2, 8, and 9.

**Fig. 1:**
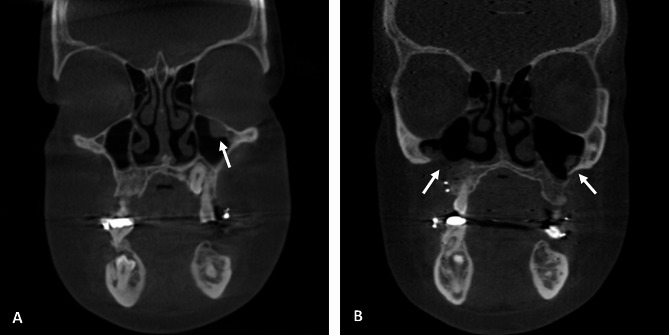
Illustrative images of Cone Beam Computed Tomography, coronal, pre- and post-operative section of the patient 9. **(A)** Pre-operative image of the left maxillary sinus with presence of radiopacity in the ceiling. **(B)** Post-operative image of the left maxillary sinus with the presence of radiopacity in the lateral wall, and of the right maxillary sinus with the presence of radiopacity on the floor. The white arrows indicate the changes found. The patient consented to have her image published

Patient 2 had a score of 1 in the right sinus both pre-operatively and post-operatively, and in the left sinus, the score increased from 0 pre-operatively to 1 post-operatively. Patient 8 had a pre-operative score of 0 in both sinuses before surgery, but a post-operative score of 1 in the left maxillary sinus. Patient 9 had a pre-operative score of 1 in the left sinus and a post-operative score of 1 in both maxillary sinuses (Fig. 1).

Patients 1, 3, 4 and 5 had the same score at both evaluations and no changes were observed on post-operative scans. Patients 6, 7 and 10 did not have post-operative scans, and it was not possible to assess whether there were any changes after OS.

**Fig. 2:**
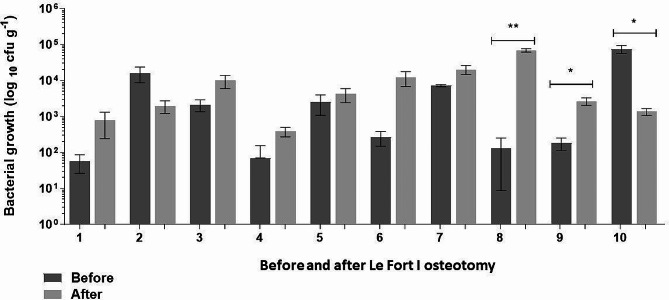
Total aerobic mesophilic bacteria counts in nasal cavity before and after Le Fort I osteotomy. Comparisons between colony-forming unit (CFU) counts in nasal cavity before and after Le Fort I osteotomy were performed using paired *t*-test (*statistically significant difference at *p* < 0.05; **p < 0.01)

The number of culturable mesophilic aerobic bacteria was determined as CFU/mL Comparing the pre- and post-operative results, a significant difference in the CFU/mL values was observed in 3 patients. An increase in the number of CFU/mL in the post-operative period was observed in patients 8 and 9, who also showed tomographic changes indicating deterioration. Patient 10 showed a decrease in the post-operative count (Table 3; Fig. 2).

**Table 3:** Total aerobic mesophilic bacteria counts expressed as colony-forming unit per millilitre in nasal cavity before and after Le Fort I osteotomy

Patient	Preoperative	Postoperative	*p*-value
P1	5.67 × 10^1^	7.83 × 10^2^	0.1502
P2	1.63 × 10^4^	1.97 × 10^3^	0.0761
P3	2.13 × 10^3^	1.01 × 10^4^	0.0970
P4	7.0 × 10^1^	3.87 × 10^2^	0.0826
P5	2.53 × 10^3^	4.2 × 10^3^	0.4652
P6	2.67 × 10^2^	1.23 × 10^4^	0.0612
P7	7.23 × 10^3^	2.03 × 10^4^	0.0502
P8	1.3 × 10^2^	6.93 × 10^4^	0.0045*
P9	1.83 × 10^2^	2.67 × 10^3^	0.0225*
P10	7.53 × 10^4^	1.37 × 10^3^	0.0218*

*Statistically significant difference at *p* < 0.05). The data was analysed by paired *t*-test

The increase in the total counts observed in patients 8 and 9 cannot be attributed to the bacteria investigated in this study, as they were not detected in the post-operative period in either of these patients (Table 4).

**Table 4:** Bacteria isolated from nasal cavity before and after Le Fort I osteotomy and their antibiotic resistance profiles

Patient	Pre- or post-operative	Bacterial isolate	Antibiotic resistance profile
1	Pre	*Staphylococcus epidermidis*	PEN, CIP*
3	Post	*Staphylococcus aureus*	AZM, ERY, PEN, CIP*
7	Pre Pre Post	*Klebsiella pneumoniae* *K. pneumoniae* *K. pneumoniae*	AMP, CFZ, CFO, CRX* AMP, CFZ, GEN, TOB, ASB, PIZ, CRX, CPM, CFO, CRO, CTX, CIP, LVX, MER, IPM, ATM AMP, CFZ, GEN, TOB, ASB, PIZ, CRX, CPM, CFO, CRO, CTX, CIP, LVX, MER, IPM, ATM
9	Pre	*S. aureus*	CIP*
		*K. pneumoniae*	AMP, CFZ, GEN, TOB, ASB, PIZ, CRX, CPM, CFO, CRO, CTX, CIP, LVX, MER, IPM, ATM

^*^Susceptible, increased exposure according to the EUCAST guidelines

PEN: Penicillin; CIP: Ciprofloxacin; AZM: Azithromycin; ERY: Erythromycin; AMP: Ampicillin; CFZ: Cefazolin; CFO Cefoxitin; CRX: Cefuroxime; GEN: Gentamicin; TOB: Tobramycin; ASB: Ampicillin-sulbactam; PIZ: Piperacillin-tazobactam; CPM: Cefepime; CRO: Ceftriaxone; LVX: Levofloxacin; MER: Meropenem; IPM: Imipenem; ATM: Aztreonam

Patient 1, who developed maxillary sinusitis in the post-operative period of OS, had one of the lowest total mesophilic bacterial counts was detected in both pre-operative and post-operative samples. *Staphylococcus epidermidis* was identified in the patient’s pre-operative sample, which was only resistant to penicillin G and showed intermediate resistance to ciprofloxacin.

The colonies that appeared as mannitol fermenters in salt mannitol agar were suspected to be *S. aureus* and were further analysed by MALDI-TOF identification. As a result, *S. aureus* was identified in two patients, one pre-operatively, which was susceptible but with increased exposure to ciprofloxacin, and susceptible to other drugs tested, and the other in the post-operative sample, which was resistant to penicillin G, erythromycin, and azithromycin, susceptible but with increased exposure to ciprofloxacin, and susceptible to other drugs tested (Table 4). None of the patients were receiving antibiotic therapy at any time during the sample collection. The drugs administered intravenously during surgery were cefazolin and tranexamic acid; during the hospitalization period, cephalothin, dipyrone, dexamethasone and oxymetazoline hydrochloride were administered intravenously; and post-operatively, after discharge from hospital, amoxicillin, ibuprofen and dipyrone were prescribed orally, as well as a rinse with chlorhexidine digluconate.

Colonies compatible with *A. baumannii* or *K. pneumoniae* on MacConkey agar were also analysed by MALDI-TOF. No isolates were identified as *A. baumannii*. However, isolates of *K. pneumoniae* were identified in two patients (7 and 9). Patient 7 had *K. pneumoniae* in both pre- and post-operative samples, with two isolates in the pre-operative sample having different antibiotic resistance profiles, one of which was the same as the post-operative isolate. The *K. pneumoniae* isolates found in this patient before and after surgery were only susceptible to amikacin, and were resistant to all other drugs tested, including all β-lactams. In patient 9, in addition to the identification of *S*. *aureus* mentioned above, *K. pneumoniae* was identified in the pre-operative sample with the same resistance profile found in the pre- and post-operative isolates from patient 7 (Table 4). None of the patients were on antibiotic therapy at the time of sampling.

## Discussion

The Le Fort I osteotomy is a common procedure used to correct bone discrepancies in individuals with dentofacial deformities. However, like any surgical procedure, there are risks, and sinusitis is a possible complication associated with Le Fort I. This association is due to the fact that the osteotomy is performed on the floor of the nasal cavity, exposing this region and potentially causing airflow problems such as obstruction in the ostiomeatal region, which can lead to sinus inflammation. In some cases, additional procedures such as septoplasty and turbinectomy may be required to prevent these complications [22]. Additionally, there is a risk of local infection and sinusopathies due to the osteotomy involving the maxillary sinus, which can be filled with a significant amount of blood and clots in the immediate post-operative period [23].

In our study, we observed a reduction in patient complaints following Le Fort I osteotomy, which is consistent with the recent review of Barone et al. [24]. Only one patient developed maxillary sinusitis after Le Fort I osteotomy, consistent with previous reports of a low incidence of this complication [22–25]. Despite its occurrence, the patient’s SNOT-22 scores and clinical evaluation suggested a transient and localized nature of the sinonasal disease, characterized by mild symptoms. Conversely, some studies have reported a relatively higher prevalence of sinusitis after Le Fort I osteotomy, accompanied by increased SNOT scores [5, 26].

This single patient who developed maxillary sinusitis after OS had one of the lowest total mesophilic bacterial counts, both pre- and post-operatively. This patient also showed improvement in the post-operative score on the SNOT-22 questionnaire, as well as the absence of hypertrophy of the palatine tonsils, scoring 1 on the Brodsky scale. The SNOT-22 questionnaire was administered and bacterial sampling was performed both before the OS and 6 months after surgery when the patient was free of sinus disease. In this patient, *S. epidermidis* was identified in the pre-operative sampling. Despite its potential to cause sinusitis, we cannot associate this clinical condition with *S. epidermidis*, as it was susceptible to drugs used in the pre- and post-operative periods. Another patient (patient 2) had worsening SNOT and Lund-Mackay scale scores, along with grade 3 tonsil hypertrophy, but no clinical diagnosis of sinusitis was made, and the bacteria under investigation were not detected. Although patients 8 and 9 were found to have worsened post-operatively in the analysis of tomographic scans and Lund-Mackay scale scores (0 to 1), they showed improvement in the SNOT-22 evaluation. This is most likely due to a mild change, since there is no total involvement of the maxillary sinuses (complete opacification of the sinuses), and changes were found in only one of the maxillary sinuses, with the patients remaining asymptomatic and even improving the symptoms of maxillary sinusitis, as indicated by the SNOT-22 questionnaire. Among the subjects, patient 10 was the sole individual with diabetes, a condition known to increase the risk of developing post-operative infections [27, 28]. Despite this, none of the bacteria investigated were detected in this patient’s post-operative nasal cavity, and a significant decrease in bacterial counts was observed in the samples analyzed. Actually, none of the bacteria detected and characterized in terms of antimicrobial susceptibility appeared to be responsible for post-operative sinus disease in the patients evaluated.

Regarding palatine tonsil hypertrophy analyses, we observed that one of the two patients who presented with hypertrophic palatine tonsils also scored high on the SNOT-22 evaluation, indicating moderate to severe problems and symptoms, both before and after surgery. This finding supports the association between the two instruments and emphasizes the importance of their use in the assessment and diagnosis of sinusopathies (Table 2). It is known that patients with swollen palatine tonsils, which can compromise the airway, may cause mechanical obstruction of the nasopharynx, blocking the airway and preventing nasal breathing, contributing to the development of sinusopathies [19, 23].

Patients found to have bacteria with the potential to cause difficult-to-treat post-operative complications were young and healthy, reducing the likelihood of sinus infection, even if they were asymptomatic carriers of multidrug-resistant bacteria. In fact, due to the elective nature of the procedure, individuals undergoing orthognathic surgery are usually healthy and immunocompetent patients, who undergo a battery of pre-operative tests and a short hospital stay. This may justify the results seen in our study, as none of the participants developed an infection after the procedure, even those who presented with isolates of *K. pneumoniae* that were extremely resistant to antibiotics. However, it is important to note that *K. pneumoniae* has been found in cases of chronic rhinosinusitis [11], so we cannot rule out this possibility in a larger sample and with longer follow-up.

In this context, it is worrying that patients 7 and 9 were colonized in the nasal cavity with *K. pneumoniae* that was already resistant to the drugs used in the pre- and post-operative period. Although, no post-operative complications due to this bacterium were observed, patient 7 remained colonized with one of the strains found pre-operatively. The presence of this bacterium in these patients suggests a community-acquired infection, not associated with the surgical or hospital procedures, which is usually the case for this microorganism [29]. This highlights the potential for the spread of multidrug-resistant bacteria in the community, even in the absence of symptoms, which could lead to infection in susceptible hosts and pose a public health threat.

The prevalence of human carriers of multidrug-resistant bacteria in the community is still uncertain, in contrast to hospital epidemiology. Several studies have investigated the incidence of these bacteria and reported a high rate of colonization of *K. pneumoniae* and *S. aureus* in healthy adults in the community [30–35]. It is noteworthy that *K. pneumoniae* has been found more frequently in the nasal cavity and respiratory tract samples compared to stool samples [36, 37], which are typically used to assess the carriage of this microorganism.

Overall, the data obtained from the SNOT-22 responses in the post-operative period, as well as the absence of tomographic alterations, demonstrated an improvement in sinusitis, a possible complication after OS, and in the quality of life of the participants. Thus, it is important to highlight that the correction of bone discrepancies by OS not only offers aesthetic benefits but also improves the patient’s airway by increasing the nasopharyngeal, oropharyngeal and laryngopharyngeal spaces, thereby improving the patient’s respiratory quality and reducing possible sinus complications. In fact, the low incidence of complications from the Le Fort I osteotomy, including sinusopathies, reinforces that the benefits outweigh the risks.

It is important to acknowledge the limitations of our study, including the small number of patients studied, the lack of long-term follow-up, and the lack of assessment of the patency of the maxillary sinus ostium, which has recently been suggested as an important factor associated with sinusitis following Le Fort I osteotomy [38]. Given these limitations, future research should prioritize larger prospective studies with standardized protocols and longer follow-up periods to comprehensively evaluate the outcomes of Le Fort I osteotomy procedures. Additionally, it is crucial to ensure adequate preparation before performing the Le Fort I osteotomy procedure, including proper diagnosis of any existing sinus disease. If a sinus disease is confirmed or suspected, the patient should be referred for an otorhinolaryngological evaluation. It is also important to evaluate the patient’s general health beforehand, as healthy carriers may harbour bacteria that are highly resistant to antibiotics, potentially causing opportunistic infections.

## Data Availability

The datasets used and analyzed during the current study are available from the corresponding author on reasonable request.

## Abbreviations

CBCT: Cone Beam Computed Tomography
OS: Orthognathic surgery
SNOT: Sino-Nasal Outcome Test
BHI: Brain Heart Infusion
CFU: Colony- forming units
MALDI-TOF: Matrix-assisted laser desorption ionization time of flight
EUCAST: European Committee on Antimicrobial Susceptibility Testing
